# Impact of Cocaine Use on Acute Ischemic Stroke Patients: Insights from Nationwide Inpatient Sample in the United States

**DOI:** 10.7759/cureus.1536

**Published:** 2017-08-02

**Authors:** Rupak Desai, Upenkumar Patel, Chintan Rupareliya, Sandeep Singh, Manan Shah, Rikinkumar S Patel, Smit Patel, Zabeen Mahuwala

**Affiliations:** 1 Research Coordinator, Atlanta Veterans Affairs Medical Center; 2 Public Health, National University; 3 Department of Neurology, University of Missouri, Columbia, Missouri; 4 Neurology, IHBAS; 5 Internal Medicine, UT Houston; 6 Department of Psychiatry, Northwell Zucker Hillside Hospital; 7 Department of Neurology, University of Connecticut Health Center; 8 Department of Neurology, University Of Kentucky College of Medicine

**Keywords:** cocaine abuse, acute ischemic stroke, cocaine dependence, stroke prevention, in-hospital outcomes, national inpatient sample, mortality

## Abstract

Cocaine is the third most common substance of abuse after cannabis and alcohol. The use of cocaine as an illicit substance is implicated as a causative factor for multisystem derangements ranging from an acute crisis to chronic complications. Vasospasm is the proposed mechanism behind adverse events resulting from cocaine abuse, acute ischemic strokes (AIS) being one of the few. Our study looked into in-hospital outcomes owing to cocaine use in the large population based study of AIS patients. Using the national inpatient sample (NIS) database from 2014 of United States of America, we identified AIS patients with cocaine use using International Classification of Disease, Ninth Revision (ICD-9) codes. We compared demographics, mortality, in-hospital outcomes and comorbidities between AIS with cocaine use cohort versus AIS without cocaine use cohort. Acute ischemic strokes (AIS) with cocaine group consisted of higher number of older patients (> 85 years) (25.6% versus 18.7%, p <0.001) and females (52.4% versus 51.0%, p <0.001). Cocaine cohort had higher incidence of valvular disorders (13.2% versus 9.7%, p <0.001), venous thromboembolism (3.5% versus 2.6%, p<0.03), vasculitis (0.9% versus 0.4%, p <0.003), sudden cardiac death (0.4% versus 0.2%, p<0.02), epilepsy (10.1% versus 7.4%, p <0.001) and major depression (13.2% versus 10.7%, p<0.007). The multivariate logistic regression analysis found cocaine use to be the major risk factor for hospitalization in AIS cohort. In-hospital mortality (odds ratio (OR)= 1.4, 95% confidence interval= 1.1-1.9, p <0.003) and the disposition to short-term hospitals (odds ratio (OR)= 2.6, 95% confidence interval = 2.1-3.3, p <0.001) were also higher in cocaine cohort. Venous thromboembolism was observed to be linked with cocaine use (OR= 1.5, 95% confidence interval= 1.0-2.1, p < 0.01) but less severely than vasculitis (OR= 3.0, 95% confidence interval= 1.6-5.8, p <0.001). Further prospective research is warranted in this direction to improve the outcomes for AIS and lessen the financial burden on the healthcare system of the United States.

## Introduction

The use of cocaine as an illicit drug surged in the United States of America between 2002 and 2007 and currently, it is the second most abused drug after cannabis and alcohol [1-3]. Among the adults, the annual prevalence of cocaine use was 1.5%, while some states reported the prevalence of 5.5% to 5.8% in the age group of 18 to 25 years. The United Nations Office on Drugs and Crime and the European Monitoring Centre for Drugs and Drug Addiction has reported an increase of cocaine use in particular parts of the world [4]. The burden of health care due to cocaine dependence was reported high in the recent studies [4]. Around 23.9 million people aged 12 years and above were reported using illicit drugs in 2012 according to data from the National Survey on Drug Use and Health [5]. A recent study from a community hospital found 2.3% people being cocaine positive during drug screening in the age group of 65 years and older. In the USA, the areas of primary concern are the one rampaged with poverty and poor education [6]

One report from the emergency department notes in the Detroit area in 2002 showed cocaine use of around 182/100,000 of the population [7]. It is not merely a problem of one country; rather it has turned into a global issue [1]. The cocaine use leads to the spectrum of multisystem derangements ranging from mild intoxication to severe complications like acute myocardial infarction, seizures and acute ischemic stroke [8]. Compared to the corresponding peer groups in the general population, cocaine users tend to have four to eight times higher mortality [9-10]. Use of cocaine is presumed to be one of the major risk factors for cerebrovascular disease, including stroke. Acute ischemic stroke (AIS) is labeled as the third leading cause of disability-adjusted life years [10].

Previous studies have reported a 19% increase in the incidence of strokes due to cocaine use in the last two decades [11]. The rise in cocaine-associated morbidity and mortality posed it as a major public health concern [11-12]. Impacts on health care economies due to stroke-related disability is devastating owing to medical cost, rehabilitation cost and cost due to loss of workforce. A direct or indirect burden of around $68.9 billion was imposed on the US healthcare owing to strokes in 2009, a major part of which was comprised of strokes as a result of illicit drugs use [2]. We aim to evaluate various factors associated with acute ischemic strokes (AIS) risk and to develop the management strategies to mitigate mortality rates within cocaine-induced stroke population.

## Materials and methods

Data source

We utilized the discharge data from the National Inpatient Sample (NIS) of the Healthcare Cost and Utilization Project (HCUP) as a source. The NIS is an all-payer dataset that includes around eight million (around 20% of the stratified sample) inpatient admissions and discharges from almost 1050 USA hospitals, excluding long term care and rehabilitation facilities. The NIS data set is unweighted and it results in the weighted estimate of the total discharge number of the US population when we apply the discharge weight to the unweighted data. We excluded the data of missing information such as age, gender, discharge condition or primary diagnosis. We used the International Classification of Diseases, Ninth Revision, Clinical Modification (ICD-9-CM) to identify admissions with the primary diagnosis of AIS. Since NIS is publicly available de-identified database from Agency for Healthcare Research and Quality (AHRQ), it does not require an approval from institutional review board (IRB). More details on the dataset content and methods of collections are accessible on the HCUP website [13].

Patient selection

We looked into the NIS database of the year 2014 to identify all patients with AIS with ICD-9-CM codes (433.01, 433.11, 433.21, 433.31, 433.81, 433.91, 434.01, 434.11, 434.91 and 436). Current use of cocaine (dependence or abuse) was identiﬁed using the ICD-9-CM codes 304.20 (cocaine dependence, unspecified), 305.62 (non-dependent cocaine use, episodic), 305.61 (non-dependent cocaine use, continuous), 304.21 (cocaine dependence, continuous) and 304.22 (cocaine dependence, episodic) [14] (Appendix).

Variables

Demographic variables examined in this study included age group (1-17, 18-44, 45-64, 65-84 years and > 85), gender (male or female), race (white, Hispanic, Asian or Pacific Islander, Native American and other) and payer source (Medicare, Medicaid, private pay, self-pay, no charge and other). Based on existing literature, we searched and defined AIS risk factors using diagnosis codes from ICD-9-CM mentioned in the appendix.

Statistical analysis

Statistical Package for the Social Science, version 22.0 (SPSS V.22.0, IBM Corp., Armonk, NY, USA) was used for all the statistical analysis. The incidence of AIS hospitalization among cocaine users and nonusers were determined by searching all available diagnosis fields for the diagnosis of AIS. Further age stratification was performed of the population into groups of 1-17, 18-44, 45-64, 65-84 years and > 85 years. Pearson’s chi-square test was used for categorical data and the independent sample T-test was used for continuous data. We used a multivariate logistic regression model to assess the AIS outcomes of cocaine use. Standard weights from HCUP were utilized to get the national weighted estimates of inpatient admissions. We defined p-value less than 0.05 (p < 0.005) as the statistical significance.

## Results

Baseline characteristics of acute ischemic strokes cohort

We identified the total of 584,115 patients with AIS by using the discharge data from NIS of the year 2014. We further stratified cohorts into a cocaine use (N=1135) and non-cocaine use (N=582980). The AIS with cocaine group consisted of higher number of older patients (> 85 years) (25.6% versus 18.7%, p <0.001), females (52.4% versus 51.0%, p <0.001) and whites (89.2% versus 70.3%, p <0.001). Cardiovascular incidences that were higher in the cocaine cohort versus non-cocaine cohort included valvular disorders (13.2% versus 9.7%, p <0.001), venous thromboembolism (3.5% versus 2.6%, p <0.03), vasculitis (0.9% versus 0.4%, p <0.003), and sudden cardiac death (0.4% versus 0.2%, p <0.02). The incidence of epilepsy was higher in cocaine cohort (10.1% versus 7.4 %, p <0.001). The incidence of major depression was higher in cocaine cohort (13.2% versus 10.7%, p <0.007). The incidences of other risk factors for AIS such as atherosclerosis, cardiac circulatory anomalies, hypertension (complicated and uncomplicated), elevated cholesterol, diabetes, transient ischemic attack (TIA), paralysis, family history of stroke, deficiency anemia, coagulopathy, disorders of fluid and electrolytes were significantly higher in the non-cocaine cohort (Table 1).

**Table 1:** Baseline characteristics of hospitalized acute ischemic stroke patients without versus with cocaine. Significant P-values ≤ 0.05 at 95% confidence interval, variables were Agency for Healthcare Research and Quality (AHRQ) comorbidity measures. Abbreviations: AIS= acute ischemic strokes, SNF= skilled nursing facility, INF= intermediate nursing facility, RA= rheumatoid arthritis, CVD= collagen vascular diseases.

Variables	AIS + NO Cocaine	AIS + Cocaine	P-value*
Unweighted admissions	116596	227	
Weighted admissions	582980	1135	
Age in years at admission			
Mean Age ± SD	69.87±15.01	72.91±14.29	<0.001
1-17	0.5%	0.0%	<0.001
18-44	4.6%	3.5%	<0.001
45-64	28.6%	23.8%	<0.001
65-84	47.5%	47.1%	<0.001
>85	18.7%	25.6%	<0.001
Died during hospitalization			
Did not die	93.1%	89.9%	<0.001
Died	6.9%	10.1%	<0.001
Disposition of Patient			
Routine	32.3%	22.5%	<0.001
To Short-term Hospitals	3.4%	11.3%	<0.001
Other (SNF, ICF, Another facility)	43.4%	37.0%	<0.001
Home Health Care	13.2%	18.1%	<0.001
Against Medical Advice (AMA)	0.7%	0.9%	<0.001
Died	6.9%	10.1%	<0.001
Discharged alive, destinations unknown	0.1%	0.0%	<0.001
Elective Vs. Non-elective Admissions			
Non-elective	93.6%	98.2%	<0.001
Elective	6.4%	1.8%	<0.001
Indicator of Sex			
Male	49.0%	47.6%	0.346
Female	51.0%	52.4%	0.346
Primary Expected Payer			
Medicare	65.7%	72.1%	<0.001
Medicaid	9.0%	9.3%	<0.001
Private including HMO	18.7%	16.4%	<0.001
Self - Pay	4.1%	1.3%	<0.001
No charge	0.4%	0.0%	<0.001
Other	2.1%	0.9%	<0.001
Race			
White	70.3%	89.2%	<0.001
Black	16.8%	6.3%	<0.001
Hispanic	7.3%	4.1%	<0.001
Asian or Pacific Islander	2.4%	0.0%	<0.001
Native American	0.5%	0.0%	<0.001
Other	2.7%	0.5%	<0.001
Co –morbidities			
RA/CVD	2.7%	4.0%	0.009
Atherosclerosis	28.7%	25.6%	0.020
Acute Myocardial Infarction	4.6%	4.8%	0.667
Arrhythmia	0.3%	0.4%	0.204
Sudden Cardiac Death	0.2%	0.4%	0.024
Cardiac Circulatory Anomalies	3.0%	1.3%	<0.001
Heart Valve Disorders	11.2%	14.1%	0.002
Vasculitis	0.4%	0.9%	0.003
Hypertension	79.8%	72.2%	<0.001
Elevated BP without Hypertension	0.2%	0.0%	0.106
Elevated Cholesterol	54.7%	42.3%	<0.001
Venous Thromboembolism	2.6%	3.5%	0.039
Viral Infection	0.6%	0.0%	0.007
Pulmonary Circulation Disorders	4.1%	6.2%	<0.001
Paralysis	10.0%	7.5%	0.005
Transient Ischemic Attacks	1.1%	0.0%	<0.001
Family History of Stroke	2.3%	0.4%	<0.001
Acute but ill-defined Cerebrovascular Disease	0.3%	0.0%	0.091
Epilepsy	7.4%	10.1%	<0.001
Other neurological disorders	5.5%	2.6%	<0.001
Depression	10.7%	13.2%	0.007
Psychoses	3.9%	2.6%	0.034
Alcohol abuse	4.5%	3.1%	0.021
Drug Abuse	3.1%	1.8%	0.011
Deficiency anemia	14.7%	12.8%	0.063
Coagulopathy	5.5%	4.0%	0.025
Metastatic cancer	1.9%	3.1%	0.005
Solid Tumor without Metastasis	1.9%	3.1%	0.005
Diabetes, uncomplicated	29.8%	26.0%	0.005
Oral contraceptive use	0.1%	0.4%	0.009
Renal failure	16.3%	11.5%	<0.001
Rhabdomyolysis	1.6%	0.4%	<0.001
Fluid and electrolyte Disorders	27.6%	20.3%	<0.001
Liver Disease	1.7%	1.3%	0.334
Obesity	11.4%	10.6%	0.356

Multivariable risk factors for acute ischemic strokes hospitalization

Table 2 shows different variables that were used in the multivariate logistic regression model to identify AIS risk factors requiring hospitalization. We found cocaine use to be the major risk factor for hospitalization. In-hospital mortality was also observed to be higher in cocaine cohort with the 95% confidence interval (CI) 1.1-1.9 (OR= 1.4, 95% CI= 1.1-1.9, p <0.003). A disposition to short-term hospitals (OR= 2.6, 95% CI= 2.1-3.3, p <0.001) and home healthcare (OR= 1.5, 95% CI= 1.2-1.9, p <0.001) was also significantly higher after adjusting for confounders. Personal history of sudden cardiac arrests (OR= 7.9, 95% CI= 3.1-20.1, p <0.001) were significantly associated with cocaine use which could be another manifestation due to potential vasospasm [8]. Venous thromboembolism was observed to be linked with cocaine use (OR= 1.5, 95% CI= 1.0-2.1, p < 0.01), but less severely than vasculitis (OR= 3.0, 95% CI= 1.6-5.8, p<0.001) (Table 2).

**Table 2:** Predictors of hospitalization in acute ischemic strokes (AIS) cocaine cohort versus acute ischemic strokes (AIS) on cocaine cohort by multivariate logistic regression. Significant P-value ≤ 0.05 at 95% and ≤ 0.01 at 99% confidence interval, variables are Agency for Healthcare Research and Quality (AHRQ) co-morbidity measures. Abbreviations: SNF= skilled nursing facility, INF= intermediate nursing facility, RA= rheumatoid arthritis CVD= collagen vascular diseases, CI= confidence interval, HMO= Health Maintenance Organization.

Variables	Odds Ratio	95% CI	99% CI	P-value*
Weekend Admissions				
Monday-Friday	Referent	Referent	Referent	
Saturday-Sunday	0.952	0.827 - 1.096	0.791 - 1.145	0.492
Disposition of Patient				
Routine	Referent	Referent	Referent	
To Short-term Hospitals	2.691	2.143 - 3.380	1.995 - 3.630	<0.001
Other (SNF, ICF, Another Facility)	0.970	0.809 - 1.164	0.764 - 1.233	0.745
Home Health Care	1.574	1.291 - 1.920	1.213 - 2.043	<0.001
Against Medical Advice (AMA)	1.370	0.719 - 2.610	0.587 - 3.196	0.338
Died	1.485	1.147 - 1.922	1.058 - 2.084	0.003
Elective versus Non-elective Admissions				
Non-elective	0.374	0.238 - 0.588	0.206 - 0.678	<0.001
Elective	Referent	Referent	Referent	
Indicator of Sex				
Male	Referent	Referent	Referent	
Female	0.966	0.849 - 1.098	0.816 - 1.144	0.597
Length of stay (cleaned)				
1 to 3 days	Referent	Referent	Referent	
4 to 6 days	0.776	0.661 - 0.910	0.629 - 0.957	0.002
7 to 9 days	1.074	0.876 - 1.316	0.822 - 1.403	0.493
10 to 12 days	1.159	0.886 - 1.515	0.815 - 1.649	0.281
≥13 days	1.252	0.988 - 1.588	0.917 - 1.711	0.063
Primary Expected Payer				
Medicare	1.311	0.694 - 2.476	0.568 - 3.024	0.405
Medicaid	1.757	0.904 - 3.415	0.733 - 4.208	0.097
Private including HMO	1.048	0.548 - 2.002	0.447 - 2.454	0.888
Self - Pay	0.635	0.262 - 1.544	0.198 - 2.040	0.317
Other	Referent	Referent	Referent	
Race				
White	9.310	3.854 - 22.488	2.921 - 29.668	<0.001
Black	3.883	1.563 - 9.648	1.174 - 12.842	0.003
Hispanic	6.792	2.688 - 17.158	2.009 - 22.958	<0.001
Other	Referent	Referent	Referent	
Median Household Income Quartile on Patient’s ZIP				
$ 1 - $ 39, 999	0.610	0.489 - 0.761	0.456 - 0.815	<0.001
$ 40, 000 - $ 50,999	0.734	0.615 - 0.876	0.582 - 0.926	<0.001
$ 51, 000 - $ 65, 999	0.977	0.843 - 1.133	0.805 - 1.186	0.759
$ 66, 000 +	Referent	Referent	Referent	
Bed Size of Hospital				
Small	1.426	1.225 - 1.662	1.167 - 1.743	<0.001
Medium	1.158	1.001 - 1.340	0.957 - 1.402	0.048
Large	Referent	Referent	Referent	
Location and Teaching Status of Hospital				
Rural	1.573	1.244 - 1.989	1.156 - 2.141	<0.001
Urban - non teaching	1.061	0.909 - 1.238	0.866 - 1.299	0.452
Urban - teaching	Referent	Referent	Referent	
Control/ownership of Hospital				
Government, non-federal	0.198	0.077 - 0.505	0.058 - 0.678	<0.001
Private, non profit	1.306	0.944 - 1.805	0.853 - 1.999	0.107
Private, invest -own	Referent	Referent	Referent	
Co –morbidities^#^				
Musculoskeletal				
RA/CVD	1.464	1.039 - 2.063	0.933 - 2.297	0.029
Connective Tissue Disorder	0.800	0.392-1.632	0.313- 2.042	0.539
Cardiovascular				
Congestive Heart Failure	1.156	0.971 - 1.376	0.919 - 1.453	0.104
Atherosclerosis	0.789	0.681 - 0.913	0.651 - 0.956	<0.001
AMI	0.841	0.622 - 1.137	0.566 - 1.249	0.259
Arrhythmia	2.744	1.093 - 6.886	0.819 - 9.194	0.032
Sudden Cardiac Death	7.950	3.135 - 20.163	2.340 - 27.013	<0.001
SupraVentricular Premature Beats	2.478	1.009 - 6.085	0.761 - 8.070	0.048
Cardiac Circulatory Anomalies	0.280	0.149 - 0.525	0.122 - 0.640	<0.001
Cardiomyopathy	1.085	0.832 - 1.415	0.766 - 1.538	0.546
Tachycardia	0.867	0.357 - 2.107	0.270 - 2.785	0.753
Heart Valve Disorders	0.679	0.363 - 1.270	0.298 - 1.546	0.226
Peripheral Vascular Disorders	1.200	0.989 - 1.456	0.931 - 1.547	0.065
Vasculitis	3.077	1.609 - 5.886	1.312- 7.217	<0.001
Hypertension	0.982	0.846 - 1.139	0.807- 1.194	0.807
Elevated Cholesterol	0.642	0.564 - 0.731	0.541 - 0.761	<0.001
Aortic and Peripheral Arterial Embolism or Thrombosis	0.682	0.278 - 1.670	0.210 - 2.214	0.402
Venous Thromboembolism	1.518	1.097 - 2.100	0.991- 2.326	0.012
Respiratory				
Chronic Pulmonary Disease	0.895	0.752 - 1.064	0.712 - 1.123	0.207
Pneumothorax (pleurisy)	0.944	0.677 - 1.316	0.610 - 1.460	0.733
Pulmonary Circulation Disorders	1.133	0.852 - 1.507	0.779 - 1.649	0.391
Neurological				
Paralysis	0.695	0.540 - 0.895	0.499 - 0.969	0.005
Family History of Stroke	0.270	0.112 - 0.654	0.085 - 0.864	0.004
Meningitis	1.951	0.786 - 4.842	0.591 - 6.443	0.150
Migraine	1.157	0.833 - 1.607	0.752 - 1.782	0.383
Epilepsy	1.563	1.266 - 1.930	1.185 - 2.062	<0.001
Other Neurological Disorders	0.469	0.320 - 0.687	0.284 - 0.775	<0.001
Psychiatry				
Depression	1.486	1.243 - 1.777	1.175 - 1.880	<0.001
Psychoses	0.832	0.575 - 1.205	0.512 - 1.354	0.331
Alcohol abuse	0.761	0.535 - 1.082	0.479 - 1.208	0.128
Drug Abuse	0.964	0.603 - 1.541	0.521 - 1.786	0.879
Hemato-oncological				
Deficiency Anemia	1.061	0.878 - 1.281	0.827 - 1.360	0.542
Chronic Blood Loss Anemia	1.071	0.437 - 2.626	0.330 - 3.480	0.880
Coagulopathy	0.811	0.593 - 1.109	0.538 - 1.224	0.190
Weight Loss	1.308	0.986 - 1.736	0.902 - 1.898	0.063
Metastatic Cancer	1.052	0.722 - 1.533	0.642 - 1.725	0.791
Solid Tumor without Metastasis	1.109	0.764 - 1.610	0.680 - 1.810	0.585
Lymphoma	0.553	0.228 - 1.343	0.173 - 1.774	0.191
Endocrinological				
Diabetes, Uncomplicated	0.981	0.847 - 1.136	0.809 - 1.190	0.799
Diabetes with Chronic Complications	1.191	0.926 - 1.531	0.856 - 1.656	0.173
Oral Contraceptive Use	8.277	3.247 - 21.097	2.420 - 28.308	<0.001
Hypothyroidism	0.749	0.617 - 0.909	0.581 - 0.966	<0.003
Renal				
Acute Renal Failure	1.539	1.278 - 1.852	1.205 - 1.964	<0.001
Rhabdomyolysis	0.224	0.092 - 0.544	0.070 - 0.718	<0.001
Fluid and Electrolyte Disorders	0.612	0.518 - 0.725	0.491 - 0.764	<0.001
Gastrointestinal				
Liver Disease	0.913	0.540 - 1.544	0.458 - 1.821	0.734
Obesity	1.255	1.027 - 1.533	0.965 - 1.633	0.026

Gender comparison of cocaine-associated mortality

Table 3 shows the gender comparison in co-morbidities associated mortality in the cocaine cohort. Higher overall mortality due to cardiac (except arrhythmia and supraventricular premature beats) causes and acute renal failure was observed in males, whereas females had increased overall mortality owing to elevated cholesterol, heart valve disorders, vasculitis, epilepsy, arrhythmia, supraventricular premature beats (SVPB), peripheral arterial thromboembolism and heart valve disorders. A similar rate of mortalities between males and females was found due to events of elevated blood pressure without hypertension (which could be owing to incidental cocaine intake) and acute cerebrovascular disease.

**Table 3:** Gender comparison in comorbidities associated mortality in acute ischemic stroke (AIS) cocaine cohort.

Comorbidities and predictors of mortality	Died		P-value
	Male	Female	
Cocaine use	0.2%	0.3%	0.160
Cardiomyopathy	11.9%	9.0%	<0.001
Acute myocardial infarction	15.5%	13.7%	<0.001
Atherosclerosis	36.0%	27.4%	<0.001
Acute renal failure	40.7%	31.3%	<0.001
Arrhythmia	0.2%	0.4%	<0.001
Supraventricular Premature Beats	0.1%	0.2%	<0.001
Sudden cardiac death	0.6%	0.3%	<0.001
Cardiac and circulatory anomalies	2.2%	1.5%	<0.001
Transient ischemic attacks	0.6%	0.5%	<0.001
Tachycardia	1.9%	1.8%	<0.001
Elevated BP without hypertension	0.1%	0.1%	<0.001
Pneumothorax and pleurisy	12.3%	9.8%	<0.001
Bronchiolitis obliterans organizing pneumonia	0.1%	0.1%	<0.001
Rhabdomyolysis	4.3%	3.0%	<0.001
Elevated cholesterol and lipids	35.1%	35.3%	<0.001
Meningitis	1.2%	0.7%	<0.001
Migraine	0.6%	1.3%	<0.001
Sickle cell disease	0.1%	0.2%	<0.001
Oral contraceptive use	0.0%	0.1%	0.472
Viral infection	1.0%	0.6%	<0.001
Heart valve disorder	11.3%	12.2%	<0.001
Vasculitis	0.4%	0.6%	<0.001
Connective tissue disorder	0.2%	1.1%	0.107
Aortic, peripheral arterial thromboembolism	1.6%	2.0%	<0.001
Acute vascular insufficiency of intestine	0.9%	0.6%	<0.001
Epilepsy	11.9%	12.2%	<0.001
Family History Stroke (cerebrovascular)	0.6%	0.7%	<0.001
Acute but ill-defined cerebrovascular disease	0.4%	0.4%	<0.001
Drug induced headache	0.0%	0.0%	0.071

Multivariable predictors of mortality

Table 4 shows the comparison of various comorbidity related mortality odds between cocaine and non-cocaine cohorts. Mortality odds owing to liver disease, metastatic cancer, cardiomyopathy, acute myocardial infarction, and epilepsy were increased in both non-cocaine and cocaine cohort. Whereas, increased odds of mortality in the non-cocaine cohort were observed due to coagulopathy, disorders of fluid and electrolyte, obesity, weight loss, solid tumor without metastasis, elevated cholesterol, pneumothorax and pleurisy and congestive heart failure. Effect on mortality due to other variables is shown in Table 4.

**Table 4:** Multivariate predictors of the mortality in acute ischemic stroke patients without cocaine use versus with cocaine use Significant P-values ≤ 0.05 at 95% and  ≤ 0.01 at 99% confidence interval, variables are Agency for Healthcare Research and Quality (AHRQ) co-morbidity measures.

Variables	No Cocaine				Cocaine			
	Odds ratio	99% Confidence Interval		P-value*	Odds ratio	99% Confidence Interval		P-value*
Co – morbidities^#^								
Deficiency anemias	0.899	0.866	0.933	<0.001	1.809	0.622	5.258	0.153
Congestive heart failure	1.540	1.487	1.596	<0.001	0.506	0.174	1.471	0.100
Chronic pulmonary disease	1.125	1.085	1.166	<0.001	1.078	0.431	2.694	0.833
Coagulopathy	1.720	1.644	1.800	<0.001	0.378	0.042	3.428	0.256
Depression	0.751	0.713	0.791	<0.001	0.191	0.048	0.753	0.002
Diabetes, uncomplicated	0.891	0.862	0.921	<0.001	0.364	0.156	0.850	0.002
Hypertension	0.725	0.701	0.749	<0.001	0.302	0.119	0.765	0.001
Hypothyroidism	1.031	0.990	1.074	0.052	1.521	0.527	4.395	0.308
Liver disease	1.210	1.107	1.322	<0.001	12.608	1.255	126.656	0.005
Fluid and electrolyte disorders	1.582	1.534	1.632	<0.001	1.046	0.430	2.546	0.896
Metastatic cancer	1.921	1.787	2.065	<0.001	4.895	1.318	18.184	0.002
Other neurological disorders	1.129	1.074	1.187	<0.001	0.461	0.064	3.314	0.312
Obesity	0.754	0.717	0.793	<0.001	0.694	0.204	2.356	0.441
Paralysis	1.494	1.437	1.553	<0.001	0.929	0.269	3.207	0.879
Peripheral vascular disorders	1.145	1.097	1.196	<0.001	3.405	1.296	8.947	0.001
Pulmonary circulation disorders	1.268	1.195	1.345	<0.001	0.295	0.075	1.157	0.021
Renal failure	1.077	1.037	1.119	<0.001	0.775	0.222	2.702	0.599
Solid tumor without metastasis	1.279	1.174	1.394	<0.001	0.534	0.075	3.785	0.409
Weight loss	1.263	1.206	1.323	<0.001	0.959	0.296	3.107	0.927
Cardiomyopathy	1.076	1.024	1.132	<0.001	3.008	0.844	10.722	0.026
Acute myocardial infarction	2.431	2.323	2.544	<0.001	7.820	2.173	28.138	<0.001
Atherosclerosis	1.071	1.037	1.106	<0.001	0.844	0.371	1.918	0.594
Tachycardia	1.950	1.746	2.179	<0.001	2.133	0.131	34.728	0.484
Elevated Cholesterol and lipid	0.558	0.541	0.575	<0.001	0.815	0.325	2.042	0.566
Pneumothorax and pleurisy	1.622	1.542	1.706	<0.001	0.263	0.048	1.449	0.044
Epilepsy	1.479	1.412	1.550	<0.001	9.322	3.721	23.355	<0.001

## Discussion

The current study found 96.5% of the AIS cocaine cohort was of the age group above 45 years with age ranging from 18 years to 85 years and above. In the age group of 85 years and above, the prevalence of AIS within the cocaine group surpassed the non-cocaine users. A plausible explanation could be that the cumulative effect of traditional risk factors, along with the long-term accumulation of chronic cocaine effect makes such population more vulnerable towards the risk of stroke [2]. The frequency of hospitalization was high among the urban hospitals set up.

A majority of the AIS patients visited the private, nonprofit hospitals. Among these, the odds of ones with cocaine use visiting the government, non-federal hospital were significantly low. The nature of the admission was nonelective understandable for most of the AIS cohort and within this cohort; it was significantly higher among the cocaine users. It could be because most of the patients are chronic cocaine abusers rather than acute. This finding is also justifiable from the older age group pattern of the study subject which is prone to the cumulative effect of the cocaine rather than acute features [2]. Odds of hospitalization among the whites were higher compared to the blacks and Hispanics in AIS cocaine cohort versus AIS non-cocaine cohort.

The mortality was found higher in blacks as compared to whites and Hispanic (21.4% versus 8.6% versus 11.1%, P=0.004 respectively). Analyzing the disposition of the patients, the short-term hospitals stay and death was significantly higher among the cocaine users. The associated higher comorbidities could be a possible explanation for such disposition in cocaine users as compared to the non-cocaine group. This finding is a serious concern because such patients could lead to a significant burden on the healthcare infrastructure. With increasing median household income, the frequency of hospitalization significantly increased among the cocaine users suggesting that the ones with low income and living in a poverty have a lower risk of using cocaine. Another reason could be that socioeconomic status is the poor predictor of the stroke among the cocaine users. Despite having high median household income among the cocaine users, their hospitalization was significantly elevated in the small and medium-sized hospitals. This could be due to the acute nature of the condition among these subjects, requiring urgent admission to any of the nearby facility.

The family history of the stroke was higher among the non-cocaine users as compared to the cocaine users suggesting that the usual mechanism of stroke development is not applicable to the cocaine user. Cocaine users quite commonly bear the traditional cardiovascular risk factors [2]. Sudden cardiac death, paroxysmal supraventricular tachycardia (PSVT), vasculitis and venous thromboembolism take higher odds of hospitalization.

Odds of hospitalization due to the paralysis were significantly higher among the non-cocaine users while seizures were high among the cocaine users. The frequency of depression was significantly higher among the cocaine abusers signifying the high morbidity among such populations. The incidence of diabetes and congestive heart failure (CHF) was severely high among the cocaine users, while the frequency of hypertension was quite similar to the other studies [2].

There was a significantly higher rate of valvular heart disease and venous thromboembolism among the cocaine users, suggesting of emboli as the major risk for stroke among these populations as compared to the non-cocaine users [15-17]. We reported the higher mortality among the cocaine users as compared to the non-cocaine users [18].

The older age of our study population could be a plausible reason, as studies with the young demographic and mild strokes have reported overall low mortality [19]. When we looked for the multivariate predictors of death in the AIS patients without cocaine use and with cocaine use, we found that epilepsy, peripheral vascular disorders, acute myocardial infarction, cardiomyopathy, tachycardia, metastatic cancer and liver diseases were associated with higher odds of mortality among cocaine users as compared to the non-cocaine users.

Hypertension and diabetes were not found to be associated with the excess mortality in an AIS cocaine cohort compared to the AIS non-cocaine cohort. Several postulated mechanisms for cocaine-induced ischemic stroke has been suggested [20-23]. Among these, the cardioembolic ischemic strokes and cardiac deaths due to chronic cocaine use have been proposed to be prominent [17, 24]. 

Study limitations

This study has undeniable limitations because of the NIS database which might have coding errors in terms of determining the diagnosis, comorbidities, and complications. Due to the inherent nature of large hospital’s database, it may over or underestimate AIS, cocaine use, comorbidities and other clinically relevant variables based on ICD-9 CM codes. This study also lacked variables such as medications and other treatment options related to AIS.

This database does not mention about the cause of death, so we cannot differentiate between in-hospital events and cause of death. It might be possible to have a selection bias in this study because of a retrospective population study. Due to large data size and getting national estimates using discharge weight as provided by NIS database, we could overcome these limitations. 

## Conclusions

To our knowledge, this is one of the very few studies demonstrating the effects of cocaine use on stroke using the nationally representative data source. Our results displaying the amplitude of the mortality in an AIS-cocaine cohort raised the question whether to consider cocaine as a risk factor in all AIS patients or not. Further research is warranted to evaluate the pathogenesis and health care burden due to cocaine-induced stroke.

## Appendices

Table 5 has the list of ICD-9-CM codes for suspected risk factors of AIS, comorbidities, in-hospital procedures and outcomes.

**Table 5:** International Classification of Disease, Ninth Revision (ICD-9) codes and the Clinical Classifications Software (CCS) codes used to identify co-morbidities, in-hospital procedures and complications. Abbreviations: ICD‐9‐CM= International Classification of Diseases, Ninth Revision, Clinical Modification; CCS= Clinical Classification Software.

Risk Factors/ Co-morbidity	Source	Codes
Acute ischemic stroke	ICD-9	433.01, 433.10, 433.11, 433.21, 433.31, 433.81, 433.91, 434.00, 434.01, 434.11, 434.91, 436
Cocaine	ICD - 9	304.20, 304.21, 304.22, 305.60 305.61, 305.62
Acute myocardial infarction	CCS	100
Peri-; endo-; and myocarditis, cardiomyopathy	CCS	97
Sudden cardiac death	ICD - 9	V12.53
Arrhythmias	ICD - 9	427.9
Supraventricular premature beats	ICD - 9	427.61
Tachycardia, unspecified	ICD - 9	785.0
Elevated blood pressure reading without diagnosis of hypertension	ICD - 9	796.2
Rhabdomyolysis	ICD - 9	728.88
Acute and unspecified renal failure	CCS	157
Bronchiolitis Obliterans organizing pneumonia	ICD - 9	516.8
Pleurisy; pneumothorax; pulmonary collapse	CCS	130
Epilepsy	CCS	83
Drug induced headache, not elsewhere classified	ICD - 9	339.3
Family Hx Stroke (cerebrovascular)	ICD - 9	V17.1
Acute vascular insufficiency of intestine	ICD – 9	557.0
Aortic and peripheral arterial embolism or thrombosis	CCS	116
Unspecified venous complication	ICD – 9	671.9
Venous thrombosis and embolism	ICD - 9	V12.51
Connective tissue diseases	CCS	210
Vasculitis	ICD - 9	447.6; 446.0–446.9
Meningitis	CCS	76
Cardiac and circulatory anomalies	CCS	213
Elevated cholesterol and lipids	CCS	53
Migraine	CCS	84
Sickle cell disease	CCS	61
Viral infection	CCS	7
Heart valve disorder	CCS	96
Atherosclerosis	CCS	114
Acute but ill-defined cerebrovascular disease	CCS	109
Transient ischemic attack	CCS	112
Oral contraceptive use	CCS	176
